# Correction: Actuated 3D microgels for single cell mechanobiology

**DOI:** 10.1039/d2lc90078e

**Published:** 2022-08-17

**Authors:** Berna Özkale, Junzhe Lou, Ece Özelçi, Alberto Elosegui-Artola, Christina M. Tringides, Angelo S. Mao, Mahmut Selman Sakar, David J. Mooney

**Affiliations:** Harvard John A. Paulson School of Engineering and Applied Sciences, Harvard University Cambridge MA 02138 USA mooneyd@seas.harvard.edu; Wyss Institute for Biologically Inspired Engineering Cambridge MA 02138 USA; Institute of Mechanical Engineering and Institute of Bioengineering, Ecole Polytechnique Fédérale de Lausanne (EPFL) CH-1015 Lausanne Switzerland selman.sakar@epfl.ch

## Abstract

Correction for ‘Actuated 3D microgels for single cell mechanobiology’ by Berna Özkale *et al.*, *Lab Chip*, 2022, **22**, 1962–1970, https://doi.org/10.1039/D2LC00203E.

The arrows in Fig. 3e were shown incorrectly in the original article. The corrected Fig. 3 is shown below. In addition the *x*-axis in Fig. 5d was not labelled correctly. The corrected Fig. 5 is shown below.

**Fig. 3:**
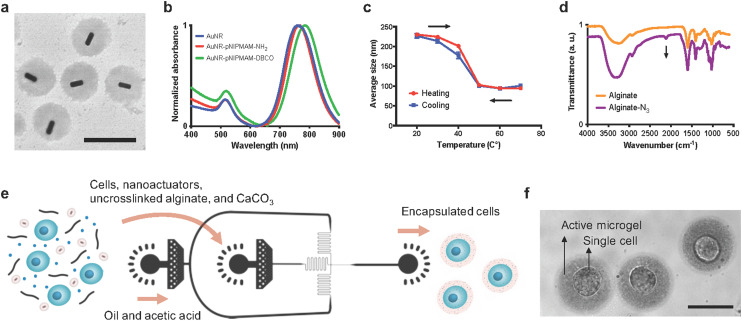
Nanoactuator synthesis and mechanically active microgel fabrication. (a) TEM image of nanoactuators. Gold nanorods appear dark surrounded by a bright appearing polymer shell. Scale bar: 500 nm. (b) UV-vis spectra of gold nanorods (AuNRs) and gold nanorod-pNIPMAM nanoactuators functionalized with either amines or DBCO. (c) Temperature induced changes in nanoactuator hydrodynamic size. Nanoactuators in an aqueous environment reversibly shrink and revert to original size when heated and cooled respectively. (d) FTIR spectra comparing unfunctionalized and azide modified alginate where arrow points to azide functional groups. (e) Schematic representation of the microfluidic encapsulation process. A pre-gel mixture of cells, alginate, calcium carbonate nanoparticles, and nanoactuators is flown through a two-channel microfluidic device. The acetic acid in the oil phase dissolves calcium carbonate and crosslinks the cell encapsulating hydrogel network as microgel droplets are formed. (f) A bright-field image showing single cell encapsulating active microgels. Scale bar: 30 μm.

**Fig. 5:**
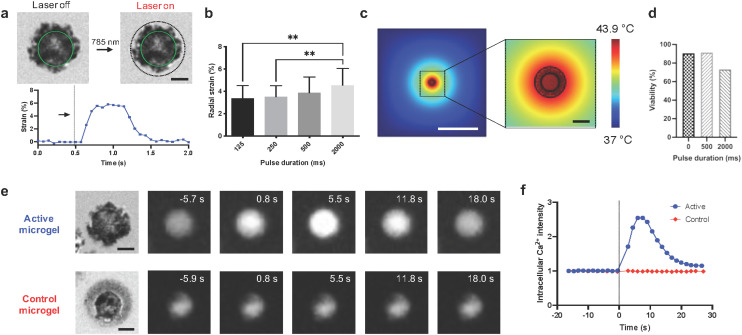
Integration of mesenchymal stem cells in active microgels and influence of isotropic compression on mechanochemical signaling in single cells. (a) Before and after actuation images of a cell encapsulated click microgel with the corresponding strain *versus* time curve, arrow shows the timepoint of actuation. A single pulse of 500 ms was applied (scale bar: 10 μm). The continuous green line indicates the cell outline in both images while the dotted black line shows the initial circumference of the active microgel. Active microgels were prepared using 18 mg ml^−1^ nanoactuator concentration with 7.1% DBCO modification. (b) Comparison of average radial strain in active microgels for 125 ms (*n* = 22), 250 ms (*n* = 54), 500 ms (*n* = 50), and 2000 ms (*n* = 34) pulse durations under 5.5 μW μm^−2^ laser power. ** indicates significant difference analyzed using one-way analysis of variance (ANOVA) followed by Šídák multiple comparison test, *p* < 0.05. (c) Simulated heat maps (scale bars: 200 μm and 20 μm respectively). (d) MSC viability before and 24 hours after a single cycle of laser actuation for 500 and 2000 ms (0 ms (*n* = 53), 500 ms (*n* = 47), 2000 ms (*n* = 41)). MSCs were encapsulated in control microgels which could be photothermally heated without exhibiting isotropic compression. (e) Series of images showing intracellular calcium intensity in active and control microgels (scale bars: 10 μm) and (f) corresponding plot presenting normalized intracellular calcium intensity over time. The radial strain in active microgels with 7.1% DBCO modification and control microgels without any DBCO was 6% and 1.3% respectively corresponding to estimated forces of 103 nN and 22 nN assuming 1 kPa Young's modulus.

The Royal Society of Chemistry apologises for these errors and any consequent inconvenience to authors and readers.

## Supplementary Material

